# Supplementary material for: Inseparable RNA binding and chromatin modification activities of a nucleosome-interacting surface in EZH2
Source: Nat Genet. 2024 May 14;56(6):1193–202. doi: 10.1038/s41588-024-01740-8 (PMC11176075; doi:10.1038/s41588-024-01740-8)
Supplement: Supplementary file 1 — Supplementary Methods and Figs. 1–7. [file 41588_2024_1740_MOESM1_ESM.pdf]

nature genetics

Supplementary information https://doi.org/10.1038/s41588-024-01740-8

Inseparable RNA binding and chromatin modification activities of a nucleosome-interacting surface in EZH2

In the format provided by the authors and unedited

Supplementary Information

- **Supplementary methods**
- **Supplementary Figures 1-7**

Supplementary Methods

For citations made in this section, see reference list in the main text.

Immunoblotting

For the immunoblotting of K562 cells, 0.5-1 million cells were resuspended in 100-200 µL 1x PBS, and then lysed using the same volume of 2x Laemmli buffer (2x (1% (v/v) SDS, 12.5% (v/v) glycerol, 35 mM Tris pH 7.5 at 25 °C, 0.001% (w/v) bromophenol blue, 5 mM $MgCl_2$, 1% (v/v) 2-mercaptoethanol) and 25 U/mL Benzonase (Merck #70746)). 50 µg of lysate per sample were loaded on either a 10% or a 12.5% gel for SDS-PAGE, and transferred to a nitrocellulose membrane (GE Life Sciences #10600002).

For the immunoblotting of mESC, cells were harvested and washed three times by 1x PBS before lysed using high salt buffer (50 mM Tris-HCl pH7.2, 300 mM NaCl, 1 mM EDTA pH7.4, 0.5% NP40, 1x protease inhibitors). Cells were then sonicated three times (10 sec on and 1 min off per cycle) with MSE Soniprep 150 Plus sonicator equipped with a 3mm Micro Tip. The lysates were then clarified at 20,000 RCF for 20 mins at 4 °C. Protein concentrations were determined using Bradford protein assay (Bio-rad # 5000006).

Membranes were incubated in blocking buffer (Thermo Scientific #37539 or 5% skim milk in 1x TBST) for 1 hour at room temperature before applying antibodies. Signal was developed using SuperSignal™ West Pico PLUS Chemiluminescent Substrate (Thermo Scientific #34580) and images were taken on a ChemiDoc™ imager. All experiments were performed in triplicates. The antibodies used for immunoblotting include: anti-actin (Sigma #A2066, 1:500), anti-EZH2 (Active Motif #39875, 1:5000), anti-H3 (Abcam #Ab1791, 1:50000), anti-H3K27me3 (Cell Signalling #9733S, 1:4000 or Active Motif #61017, 1:2500), anti-CBX7 (Abcam #21873, 1:1000), anti-SUZ12 (Santa Cruz Biotechnology #sc-271325, 1:200), anti-EZH1 (Cell Signalling #42088, 1:1000), anti-H3K27me1 (Merck #07-448, 1:1000), anti-H3K27me2 (Abcam #24684, 1:2000), anti-GAPDH (Proteintech #10494-1-AP, 1:4000), anti-mouse HRP-conjugated (Jackson Immuno-Research #715-035-150, 1:5000) and anti-rabbit HRP-conjugated (Santa Cruz Biotechnology #sc-2357, 1:5000).

CUT&Tag

CUT&Tag experiments were performed according to the protocol that was previously described[31]. In short, nuclei were isolated from harvested cells and lightly crosslinked using formaldehyde to a final concentration of 0.1% prior to slowly being frozen using Mr. Frosty container (Thermo Fisher Scientific #5100-0001). An aliquot of 200,000 crosslinked nuclei was conjugated to 10 µL of activated Concanavalin A coated beads (Bangs laboratories #BP531) per sample. Subsequently, samples were incubated with 1 µL primary antibody (H3K27me3: Cell Signaling Technology #9733; H3K27ac: Abcam #4729; Rabbit IgG control: Cell Signaling Technology #2729) overnight at 4 °C in 50 µL antibody buffer (20 mM HEPES pH 7.5, 150 mM NaCl, 0.5 mM spermidine, 2 mM EDTA, 0.1% BSA, 1x protease inhibitors). Incubation of the secondary antibody (Guinea pig anti-rabbit antibody: Antibodies online #ABIN101961) was performed at RT for 1h in 100 µL Wash buffer (20 mM HEPES pH 7.5, 150 mM NaCl, 0.5 mM spermidine, 1x protease inhibitors). After three washes using Wash buffer, samples were

incubated with pA-Tn5 adapter complex at RT for 1h in 100 µL 300-Wash buffer (20 mM HEPES pH 7.5, 300 mM NaCl, 0.5 mM spermidine, 1x protease inhibitors). Tagmentation was done at 37 °C for 1h in 300 µL Tagmentation buffer (300-Wash buffer supplemented with the final concentration of 10 mM $MgCl_2$). Reversing the crosslinks was done by adding 10 µL of 0.5 M EDTA, 3 µL of 10% SDS and 2.5 µL of 20 mg/mL Proteinase K and incubating 1h at 55 °C. DNA was then extracted using Phenol/Chloroform and precipitated using 100% ethanol. PCR was performed using NEBNext HiFi 2x PCR Master Mix (NEB #M0541), with indexed primers that were described previously[49] and a total of 13 cycles. PCR products were purified using 1.3x volume of Ampure XP bead slurry and eluted in 25 µL of 10 mM Tris-HCl pH 8.0. DNA was sequenced using Illumina Hiseq PE 150 or Novaseq PE150 Sequencing.

The analysis of CUT&Tag data followed the code outlined in a published online protocol[50,51] with some modifications. First, the --local flag was used for Bowtie2 alignment in order to remove adapter sequences. Second, potential duplicates were removed from the BAM files using the MarkDuplicates function from the Picard toolkit (https://broadinstitute.github.io/picard/), with these BAM files were then used to calculate scaling factors for H3K27me3 and H3K27ac datasets using the ChIPseqSpikeInFree R package[52]. These scaling factors were used to generate bigwig files for heatmap, enrichment profiles, and genome browser tracks. Heatmaps and enrichment profiles were generated using the command-line tool deepTools[53] and genome tracks were created using pyGenomeTracks[54,55]. Correlation matrices were generated following the cited protocol and the plots were generated using the R package pheatmap (version 1: 726)[56].

CUT&Tag peaks of H3K27me3 were calculated using SEACR[57] as previously described[50,51]. The consensus peaks of all 3 replicates were found using the findOverlapsOfPeaks() function within the ChIPpeakAnno R package[58]. This function was also used to find overlaps between mutants and the wild type. Peaks were associated to genes by annotating them to windows of 1000 bp downstream to 1000 bp upstream of transcription start sites (TSSs). Peaks were associated with differentially expressed genes using gene names.

RNA-seq

RNA was extracted from 1 million cells using RNeasy kit (QIAGEN #74004) as per manufacturer's instructions. cDNA libraries were constructed using NEBNext® Ultra™ RNA Library Prep Kit for Illumina®. cDNA libraries were pooled and sequenced using Illumina Novaseq instrument with a 2x150bp paired-end configuration.

RNA-seq reads were obtained in FASTQ format and were quantified against the hg38 transcriptome index using Salmon (version 0.14.1) with the --gcBias flag[59]. These transcript-level quantifications were then imported into R using the R packages DESeq2[60] and tximport[61]. Batch effect removal was done with limma, using the function removeBatchEffect()[62]. Next, this normalized data was visualized using a principal component analysis (PCA) plot using the DESeq2 function plotPCA()[60].

Differential expression analysis was done using DESeq2[60]. Genes were considered differentially expressed if identified under a Benjamini-Hochberg adjusted p-value of 0.01. Gene ontology (GO) enrichment analysis and visualisation was done using the R package

clusterProfiler[63]. GO terms were considered if obtained under a Benjamini-Hochberg adjusted p-value of 0.01.

Quantitative ChIP-Seq (ChIP-Rx)

K562 were collected, counted and washed once with PBS before crosslinking for 10 minutes with PBS containing 1% formaldehyde (Sigma). Cells were crosslinked in 15 mL falcon tubes at a density of ~$5x10^6$ cells/mL. Crosslinking was quenched with 0.125 M Glycine prior to two PBS washes. Crosslinked cells were then snap-frozen on liquid nitrogen and stored at -80 ºC before proceeding with the rest of the ChIP assay. Crosslinked K562 cells were thawed on ice and lysed in 4 mL of SDS-Lysis buffer (100 mM NaCl, 50 mM Tris-HCl pH 8.1, 5 mM EDTA pH 8.0, 0.5% SDS and 1X protease inhibitor cocktail (Sigma #P8340)). Chromatin/nuclei were pelleted by centrifugation at 1200 RPM for 6 mins at room temperature. The supernatant was then discarded, and the nuclear/chromatin pellet was resuspended in 2 mL of ChIP buffer, obtained by combining 1.33 ml of SDS-Lysis buffer with 0.67 ml of Triton Dilution buffer (100 mM Tris-HCl pH 8.6, 100mM NaCl, 5 mM EDTA pH 8.0, 5% Triton X-100, 1X protease inhibitor cocktail).

To allow for a quantitative ChIP-seq (ChIP-Rx)[64], with respect to a reference genome, a total of 10% mESC (WT BRUC4 mESC) was added to each K562 chromatin lysate. Spike in percentage was calculated based on cell number and was added prior to sonication. Chromatin was sheared to approximately 200 bp to 500 bp fragments by sonication using a Bioruptor Plus (Diagenode) at high power. Total “on” sonication time was 15 min with 30 sec of “on” and 30 sec of “off” pulses. Sonicated chromatin was incubated overnight with the antibodies while rotating at 4 ºC. Prior to overnight incubation with antibodies, an input sample was taken (1%). 10 million cells were used per ChIP. In the following morning, samples were clarified by centrifugation at 20,000 g for 20 min at 4 ºC. Following clarification, the chromatin was incubated for 3 hours with Protein G Dynabeads (ThermoFisher #10004D), where 50 µL beads were used per ChIP. After incubation, the beads were washed three times in Mixed Micelle Buffer (150 mM NaCl, 20 mM Tris-HCl pH 8.1, 5 mM EDTA pH 8.0, 5.2% Sucrose,1% Triton X-100, 0.2% SDS), twice with Buffer 500 (0.1% Sodium Deoxycholate, 1 mM EDTA pH 8.0, 50 mM HEPES pH 7.5, 1% TritonX-100), twice with LiCl detergent wash (0.5% Sodium Deoxycholate, 1 mM EDTA pH 8.0, 250 mM LiCl, 0.5% NP-40,10 mM Tris-HCl pH 8.0) and finally, one wash with TE. All washes were performed for 5 min with rotating at 4 ºC. Immunoprecipitated material was eluted from the beads with 100 µL elution buffer (0.1 M NaHCO3, 1% SDS) while shaking for 1 hour at 65 ºC. The supernatant was retained and incubated overnight at 65 ºC while shaking to reverse the crosslinks. The eluted material was then subjected to RNase A (DNase and protease-free Thermo Fisher Scientific #EN0531) and Proteinase K (ThermoFisher #EO0491) treatments prior to DNA clean up (QIAGEN MinElute PCR Purification Kit #28004).

Antibodies used for ChIP: Anti-EZH2 (Cell Signalling #5246), 6.3 µg per ChIP. Anti-SUZ12 (Cell Signalling #3737), 0.5 µg per ChIP. Anti-H3K27me3 (Cell Signalling #35861SF), 5 µg per ChIP.

Following the ChIP experiment, the precipitated DNA is already including the spike in reference (see above) and is quantified using the Qubit dsDNA High Sensitivity Assay Kit (ThermoFisher #Q32854). A total of 1-5 ng of DNA from each ChIP experiment was used for

library preparation using the NEBNext Ultra II DNA Library Kit for Illumina (NEB #E7645) and NEBNext Multiplex Oligos for Illumina (NEB Set 1 #E7335 & Set 2 #E7500). Following adaptor ligation, DNA was PCR amplified for 5-10 cycles, depending on the amount of the input DNA. The DNA was purified using NEBNext Sample Purification Beads (NEB #E7767S). The quality and size distributions of DNA libraries were verified on a High Sensitivity D1000 Screen Tape (Agilent). The resulting libraries were then used for cluster generation and sequencing using an Illumina based sequencer with 150 bp paired-end read length (Azenta).

For the bioinformatic analysis of ChIP-Rx datasets, chromosome names for the mouse genome were modified with the prefix 'mm10_' and a metagenome was created by concatenating the human and mouse reference genomes (hg38 and mm10, respectively) before indexing with Bowtie2[65] as described previously[64]. Reads were aligned to the metagenome using Bowtie2 with default parameters[65]. Non-unique read alignments were filtered out using SAMtools[66] to exclude those with an alignment quality of <2, and the mm10_ prefix appended to chromosome names was used to separate reads as aligned to the reference human or spike-in mouse genomes. SAMtools was used to convert SAM files to BAM files and to remove duplicate aligned reads. Spike-in normalisation factors were calculated for each ChIP using the formula for normalised reference-adjusted reads per million (RRPM) as described[64] (1 per million spike-in reads). BigWig files were generated using the bamCoverage tool from the deepTools suite (version 3.3.0)[67] with a bin size of 10 and data were subsequently visualised as ChIP-Rx normalised tracks using the IGV genome browser. Transcription start sites (TSS) for the hg38 human genome build were defined and annotated, to include TSS of all unique protein coding genes, including 5 kb upstream and downstream to them (n=17,139; downloaded from ENSEMBL biomart). Average enrichment profiles were generated with this TSS annotation file using the computeMatrix and plotProfile tools from the deepTools suite[67].

Cellular fractionations

K562 cells were lysed in 400 µL pre-extraction buffer (20 mM HEPES pH 7.2, 0.5% Triton X-100, 50 mM NaCl, 3 mM $MgCl_2$, 300 mM Sucrose, 1X protease inhibitor cocktail) and incubated on ice for 30 minutes. 200 µL of suspension was removed and labelled "Total extract" and 200 µL of 2X SDS-PAGE sample buffer was added. The remaining lysate was clarified at 20,817 g in a 4°C centrifuge for 10 minutes. Supernatant was kept and labelled "Soluble" and 200 µL of 2X SDS-PAGE sample buffer was added. The insoluble pellet was washed once in 1mL of pre-extraction buffer before resuspension in 200 µL of pre-extraction buffer and 200 µL of 2X SDS-PAGE sample buffer. All samples were boiled at 99°C for 5 minutes and sonicated 3 times for 15 s at 10% amplitude in a MSE Soniprep 150 Plus sonicator prior to separation by SDS-PAGE and western blotting.

Co-Immunoprecipitation (co-IP)

On day 0, K562 cells were infected with lentiviruses as described above per CRISPR/Cas9 knockout of EZH2 and rescue experiments. After day 7 sorting, cells were seeded in a 15 cm dish, harvested on day 14, and a running passage was continued for additional repeats. Cells were trypsinized and washed with PBS at 4 °C and harvested at 500 RCF for 5 min. The cell pellets were lysed in 300 µL cold buffer C (20 mM HEPES pH 7.9, 420 mM KCl, 1.5 mM $MgCl_2$, 25% Glycerol, freshly added 10 mM DTT and 1X protease inhibitor cocktail (Sigma

#4693132001)) for 15 minutes, and then 540 µL of buffer D was added to reduce the salt concentration (20 mM HEPES pH 7.9, 20% Glycerol, freshly added 1X protease inhibitor cocktail), as well as 0.85 µL of benzonase (Merck #70746). Concentrations were checked by nanodrop, and 80 µL of input was taken and mixed with LDS (final concentration: 1% (v/v) SDS, 12.5% (v/v) glycerol, 35 mM Tris pH 7.5 at 25 °C, 0.001% (w/v) bromophenol blue, 5 mM $MgCl_2$, 1% (v/v) 2-mercaptoethanol) and 25 U/mL Benzonase (Merck #70746)) and boiled for 5 min at 95 °C.

1.5 µg of anti-SUZ12 antibody (Cell Signalling #3737S) or 1.5 µg control IgG (Cell Signalling #3900S) was added to the lysate and incubated on a rotator for 2 h at 4 °C. 50 µl of Dynabeads Protein G beads (Thermo Fisher Scientific #10003D) prewashed with PBS was added and rotated at 4 °C for an additional hour. Beads were washed with 1 ml lysis buffer (1:1 buffers C:D) once, then once with 1 ml of each of the Wash Buffers 2, 3 and 4 (Wash Buffer 2: 50 mM Tris-HCl pH 8.0, 150 mM NaCl; Wash Buffer 3: 50 mM Tris-HCl pH 8.0, 450 mM NaCl; Wash Buffer 4: 50 mM Tris-HCl pH 8.0). Beads were resuspended in a 100 µL of 1x LDS buffer and heated to 95 °C for 10 minutes. Lysate was loaded on a 10% SDS-PAGE gel and then western blot was carried out as described above, with the exception that a titer of 1:250 of the SUZ12 antibody was used.

Supplementary Figures

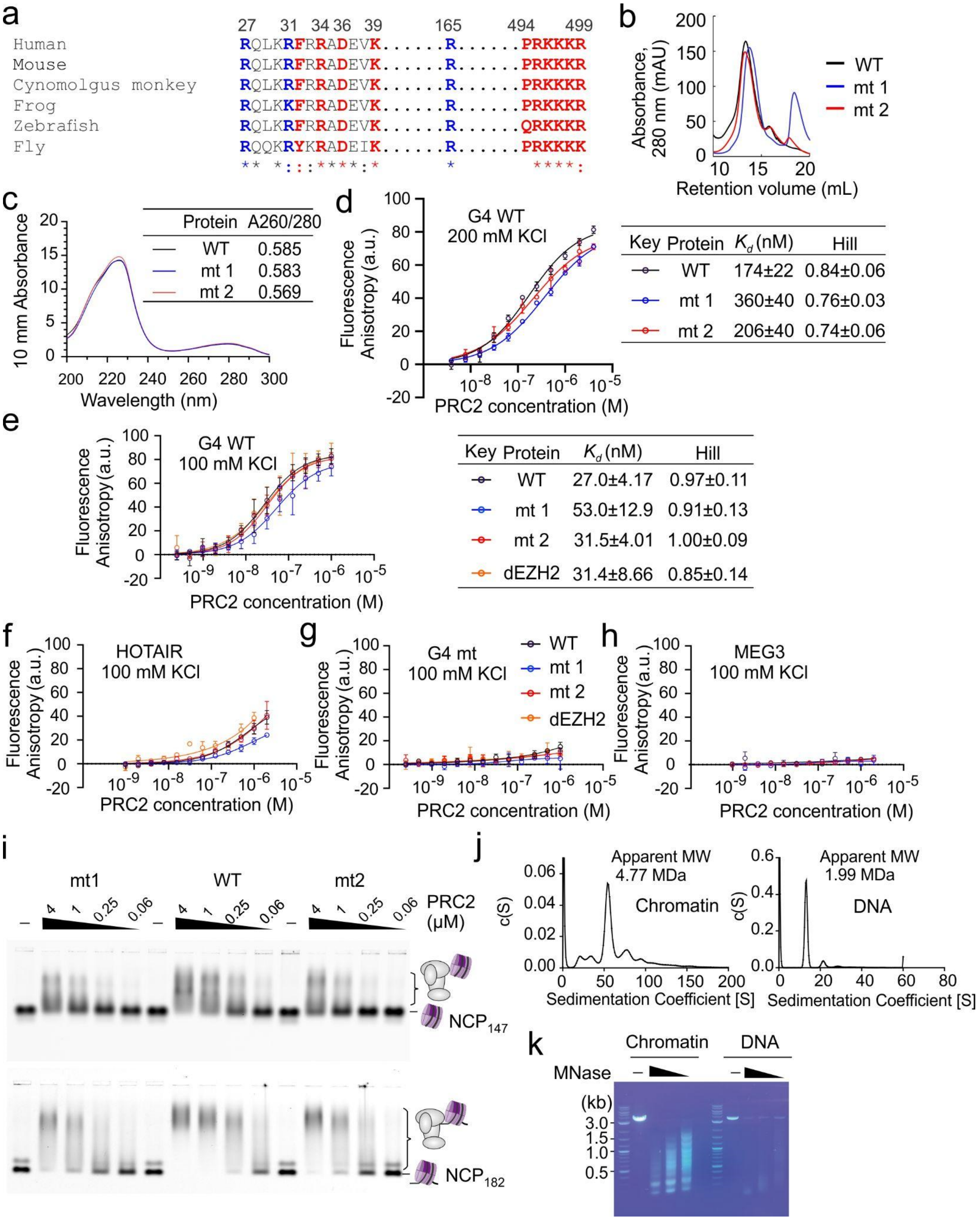

Supplementary Figure 1. An RNA-binding surface in EZH2 is required for the methylation of naïve chromatin in an RNA-independent manner.

a. Sequence alignment of EZH2 across multiple species with the mutated regions presented. The amino acids of mt1 and mt2 are coloured using the same colour code as in Fig. 1. **b.** Gel filtration chromatography (Superose 6 Increase 10/300 GL column) of the PRC2 wild-type and mutants, as indicated. **c.** The ratio of absorbance at 260 nm and 280 nm of the purified PRC2 complexes were determined using a spectrophotometer. **d.** Fluorescence

anisotropy performed to quantify the binding affinity of PRC2 wild-type and mutants for the 3′ fluorescein labeled G4 24 RNA $(UUAGGG)_4$. **e-h.** Fluorescence anisotropy performed to quantify the binding affinities of PRC2 complexes for the indicated RNA. For all the fluorescence anisotropy experiments in d-h, data represent the mean of three independent experiments that were carried out on different days and error bars represent standard deviation. Fluorescence anisotropy values are in arbitrary units (a.u.). **i.** EMSA used to assess the affinity of the PRC2 wild-type and mutants for H2A-Cy5-labelled nucleosome core particle with (NCP_{182}) or without (NCP_{147}) linker DNA. **j.** Analytical ultracentrifugation (AUC) analysis of the chromatinized array (left) and DNA (right), with the apparent molecular weight is indicated. Three replicates were carried out on different days with similar results. **k.** MNase digested fragments of the chromatinized array and DNA are visualised by agarose gel. This analysis was carried out once.

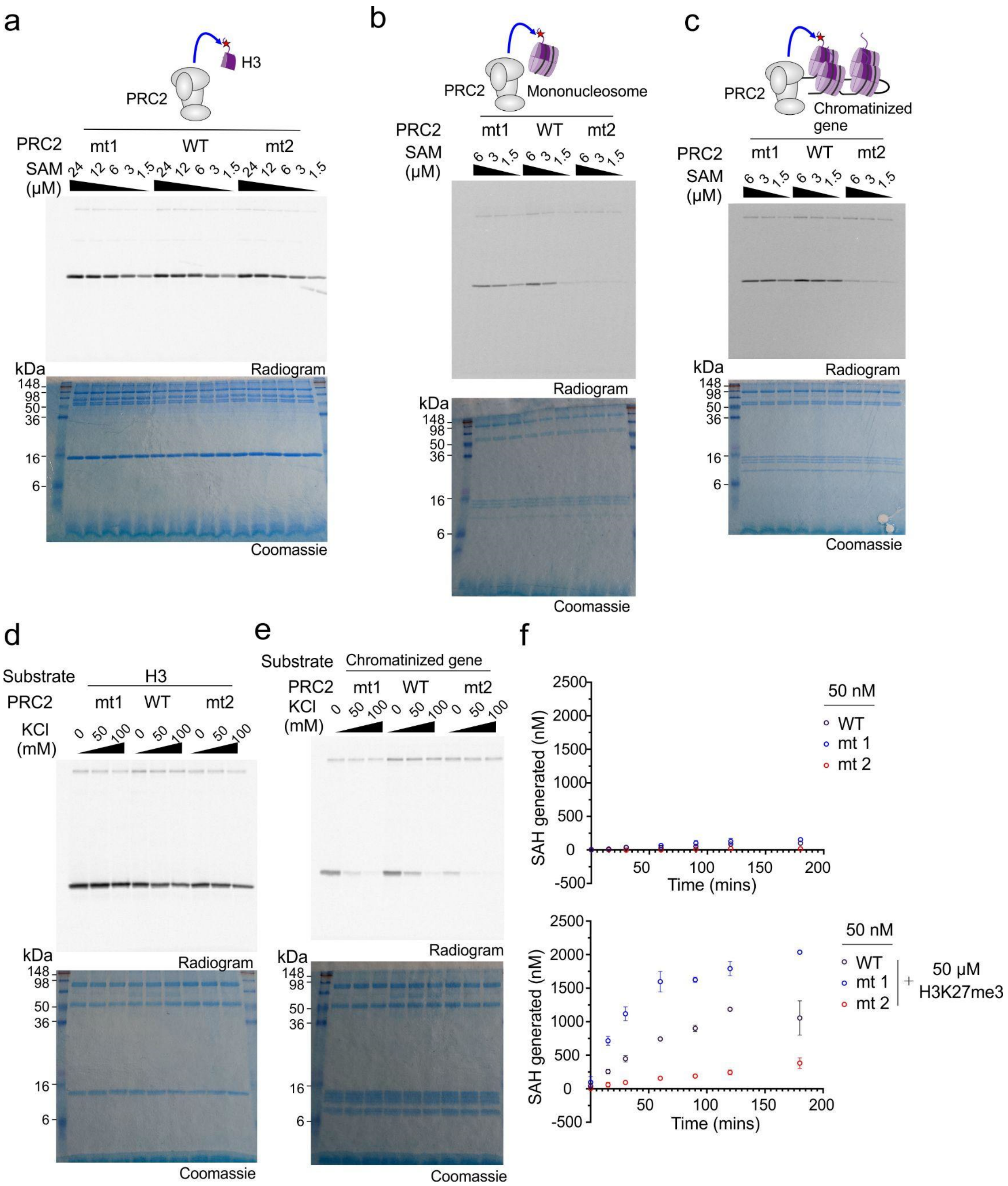

Supplementary Figure 2. An RNA-binding surface in EZH2 is required for the methylation of naïve chromatin in an RNA-independent manner.

a. The full radiogram and the corresponding uncropped Coomassie blue-stained SDS-PAGE as shown in the left panel of Fig. 1d. **b.** HMTase assay performed with 0.6 μM NCP_{147} as substrates, using 0.6 μM wildtype or mutant PRC2 complexes, as indicated. **c.** The full radiogram and the corresponding uncropped Coomassie blue-stained SDS-PAGE as shown in the right panel of Fig. 1d. **d,e.** The full radiogram and the corresponding uncropped Coomassie blue-stained SDS-PAGE as shown in the Figure 1f. **f.** Progress curves of 50 nM PRC2 complexes as indicated in the presence or absence of 50 μM allosteric effector peptide H3K27me3 on 1200 nM naïve chromatin substrate, with the chromatin concentration defined as NCP molar equivalent. Produced SAH concentrations were quantified using the

MTase-Glo™ Methyltransferase Assay (Promega) and are indicated. Presented are the means, and the error bar represents the standard deviation. In all the experiments in this figure, three independent replicates were carried out in different days.

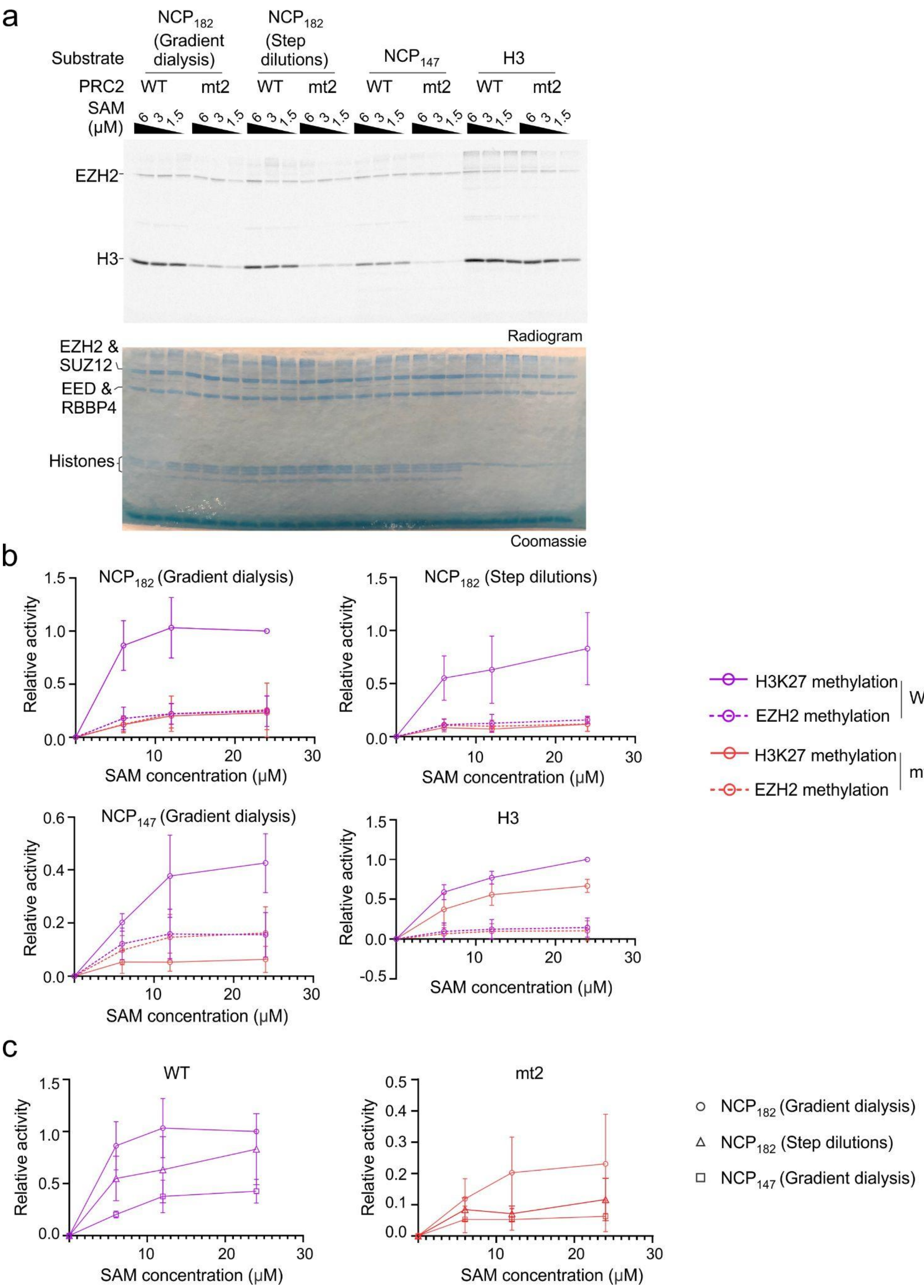

Supplementary Figure 3. The RNA-binding surface of EZH2, but not its RNA-binding activity, is required for maintaining global H3K27me3 in lineage-committed cells.

a. HMTase assay performed with indicated substrates and PRC2 complexes. Substrate concentration was 1.2 μM H3 histone or 0.6 μM mononucleosomes with (NCP_{182}) or without (NCP_{147}) linker DNA and enzyme concentration was 0.6 μM. Three independent replicates

were carried out on different days with similar results. **b.** The plots represent the relative HMTase activities of PRC2 wildtype and mutant from panel (a)**.** For all nucleosomal substrates, relative HMTase activities are obtained by a normalisation to the wild-type PRC2 activity towards NCP_{182} (gradient dialysis). For the H3 substrate, activities were normalised to the activity of the wildtype PRC2 against histone H3. Solid lines indicate PRC2 activity on the H3 substrate and dash lines indicate PRC2 activity on EZH2 (i.e. automethylation). **c.** The plots represent the relative HMTase activities of PRC2 complexes from panel (a), except that each plot directly compares HMTase activities of a given enzyme on different substrates. The activities in each plot were normalised to the HMTase activities of the corresponding PRC2 enzyme towards H3 substrates. In panels (b-c), presented are means from three independent experiments that were carried out on different days, and error bars represent standard deviation.

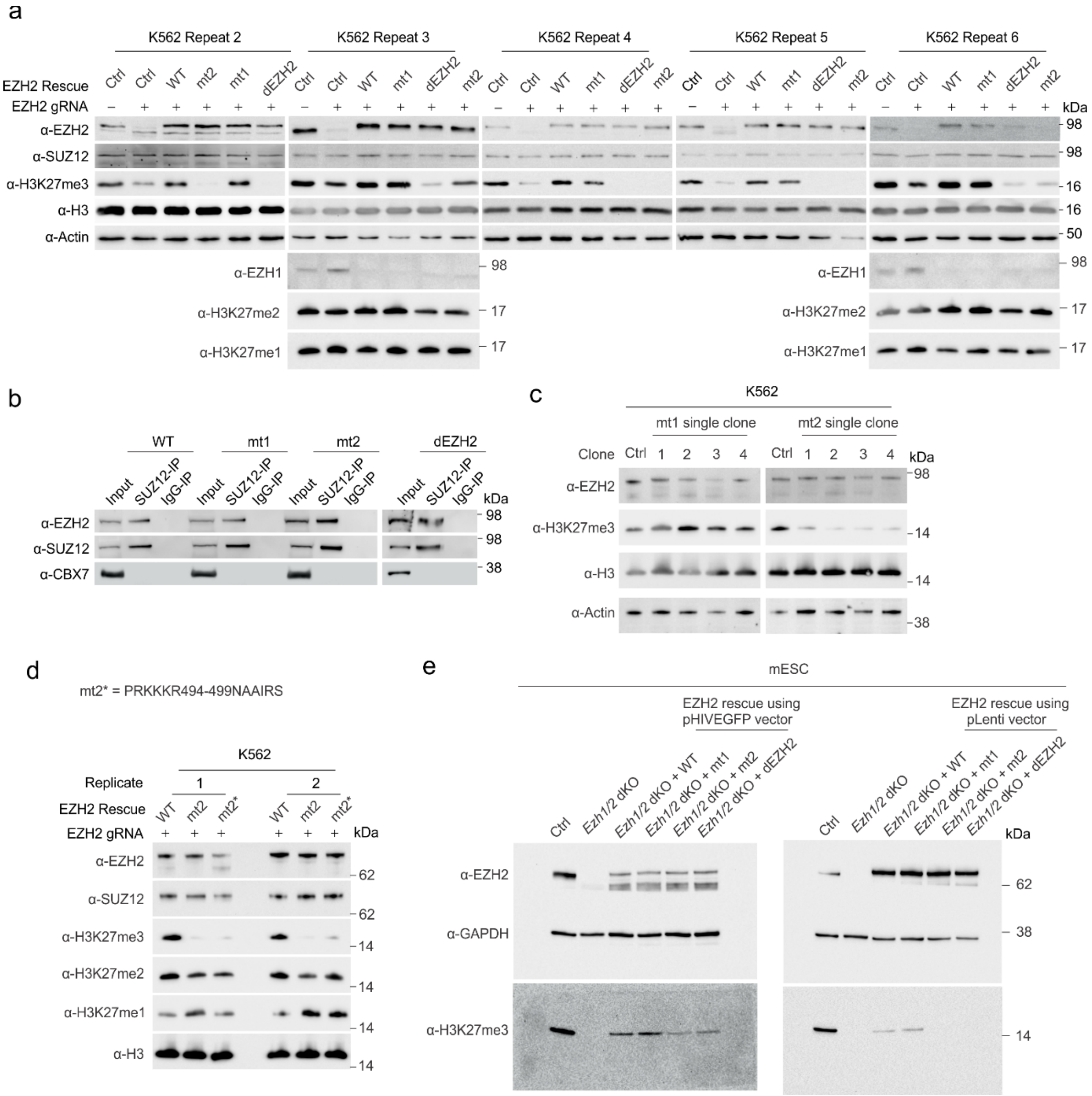

Supplementary Figure 4. The RNA-binding surface of EZH2, but not its RNA-binding activity, is required for maintaining global H3K27me3 in lineage-committed cells.

a. Independent replicates of western blot analysis of whole-cell lysates of the indicated EZH2 knockout with rescued K562 cell lines. These five replicates were carried out on five different days and are presented in addition to the one replicate in Fig. 2b (i.e. a total of six independent replicates), with the same legend applied herein. **b.** Western blot for anti-IgG or anti-SUZ12 co-immunoprecipitated (co-IP) materials from the control (Ctrl) K562 cell line and the EZH2 knockout with rescued K562 cell lines (EZH2 WT, mt1, mt2 and dEZH2). Two replicates were carried out with similar results. **c.** Western blot for the selected clones of the EZH2 knockout with rescued K562 cell lines (EZH2 mt1 and mt2), was carried out once on multiple clones as indicated. **d.** Western blot for the two independent replicates of the indicated EZH2 knockout with rescued K562 cell lines. **e.** Western blot analysis of whole-cell lysates from the indicated mouse embryonic stem cell lines, where *Ezh1* and *Ezh2* were knocked out and the indicated EZH2 rescues were carried out. The “Ctrl” line represents the parental *Ezh1* knockout mESC line, which has been used to generate the *Ezh1* and *Ezh2* double knockout line (Ezh1/2 dKO). Presented are results from two independent

experiments, where different types of lentiviral expression vectors were used to rescue EZH2 in the Ezh1/2 dKO mESC line (pHIV-EGFP on the left and pLenti on the right).

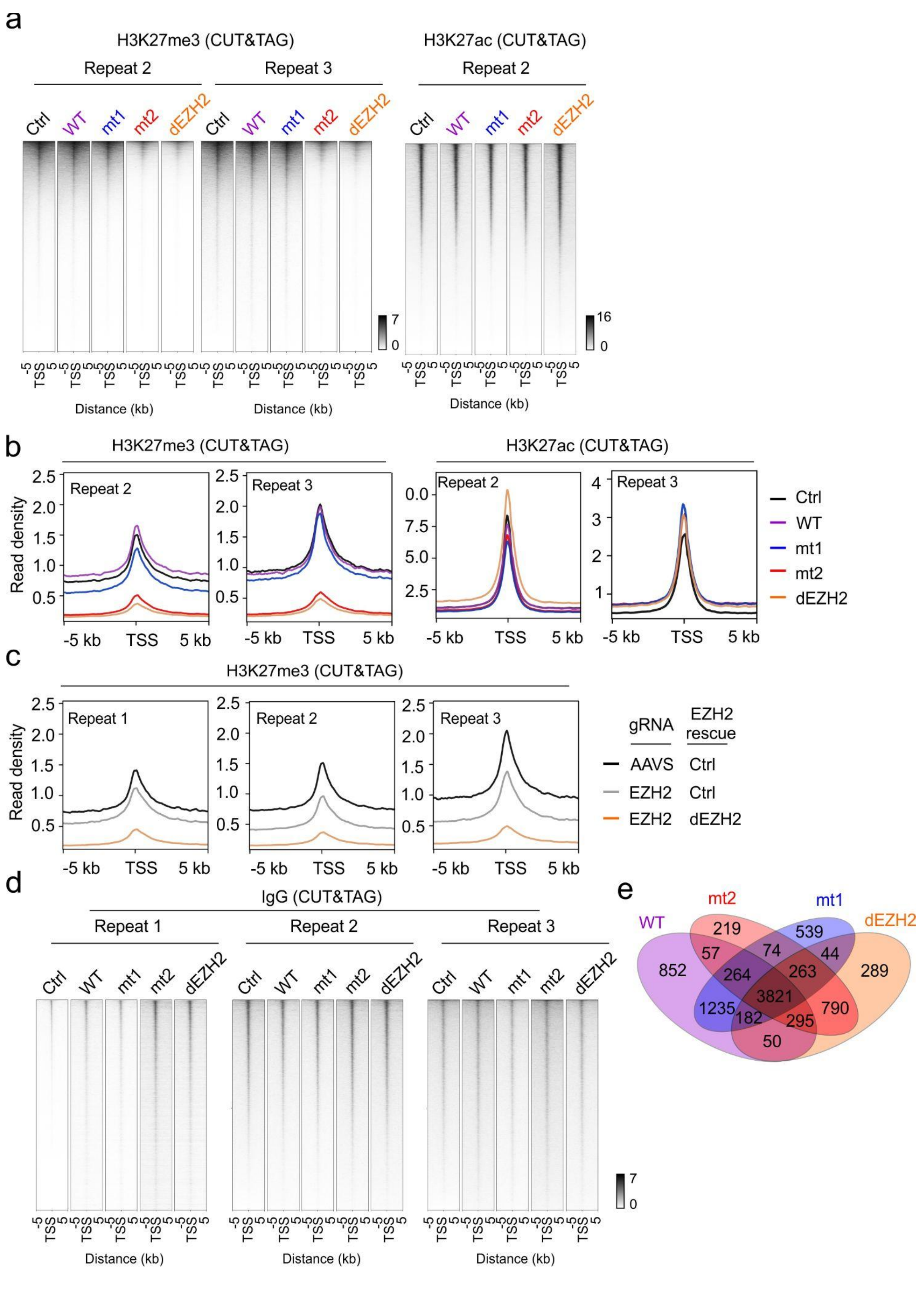

Supplementary Figure 5. The RNA-binding surface of EZH2, but not its RNA-binding activity, is required for maintaining H3K27me3 at repressed genes.

a. Heatmap of H3K27me3 and H3K27ac CUT&TAG signals 5kb upstream and downstream from TSS (see Fig. 3b for repeat 1). **b.** Enrichment profiles of the average distribution of

repeats 2 and 3 of the H3K27me3 and H3K27ac CUT&TAG over 5kb upstream and downstream from TSS. **c.** Enrichment profiles of H3K27me3 CUT&Tag for the indicated cell lines over 5 kb upstream and downstream from TSS**. d.** Heatmaps representing the IgG CUT&TAG signals 5 kb upstream and downstream from TSS. **e.** Venn diagram showing the overlap between the number of H3K27me3 CUT&Tag consensus peaks that were identified in all replicates of the indicated EZH2 knockout with rescued K562 lines (EZH2 WT, mt1, mt2 and dEZH2). All independent replicates were carried out on different days.

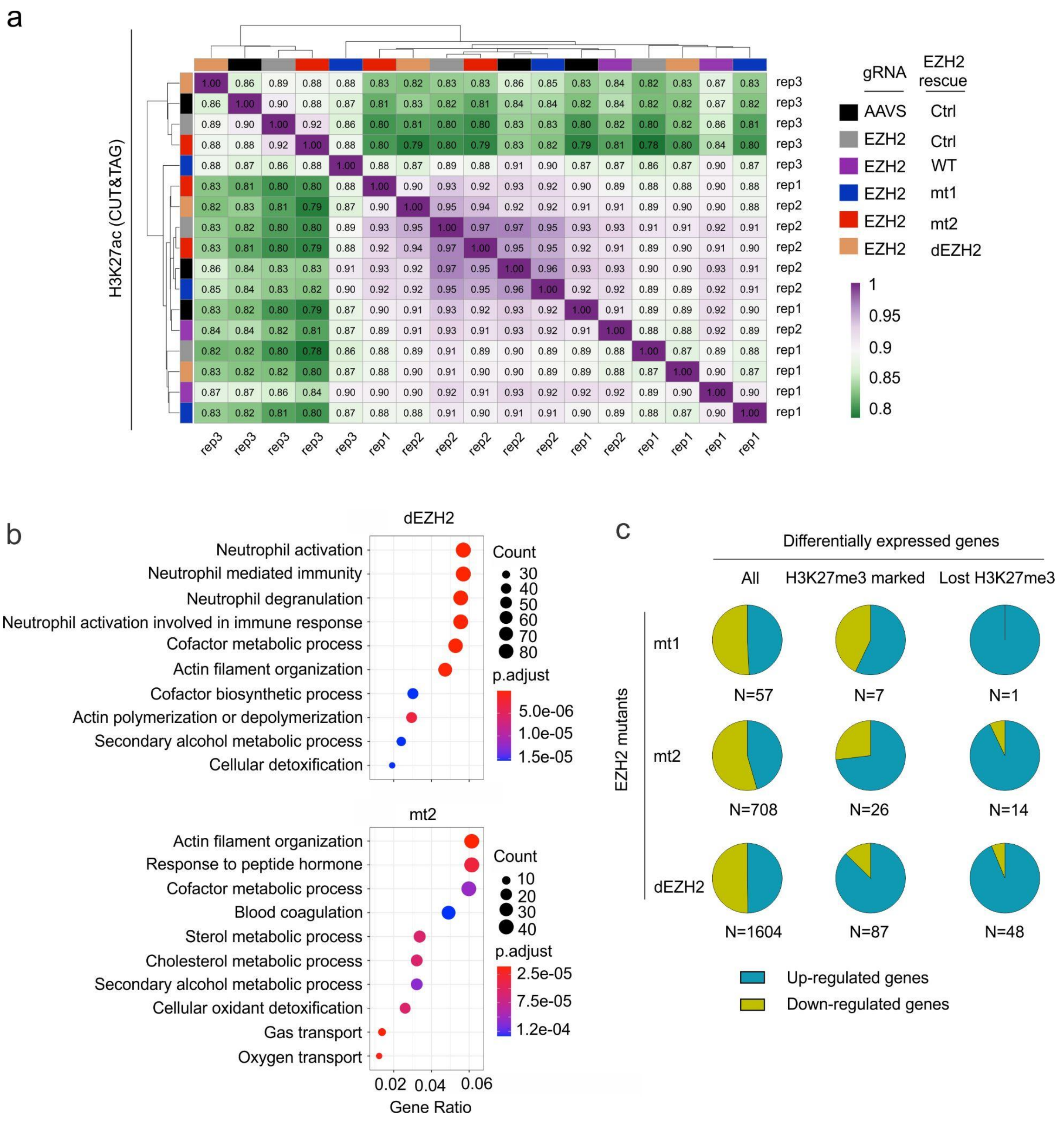

Supplementary Figure 6. An RNA-binding surface in EZH2, but not its RNA-binding activity, is required for maintaining transcription programs in lineage-committed cells. **a.** Unsupervised clustering of genome-wide correlation between H3K27ac CUT&TAG data. Values in boxes represent Pearson's correlation coefficients between corresponding samples. Samples are represented by different colours at the top and the left of the plot, with the corresponding colour code indicated at the right of the figure. **b.** Gene Ontology (GO) terms that were obtained from GO analysis of differentially expressed genes in the indicated EZH2 mutant-rescue line with respect to the wild type-rescue line, where p.adjust represents Benjamini-Hochberg adjusted p-value. **c.** Pie charts represent the number of differentially expressed genes in the indicated EZH2 mutant-rescue K562 lines, where differential expression analysis was carried out with respect to the wild type EZH2-rescue line. The group "All" represents all the differentially expressed genes for a given EZH2 mutant line, disregarding if they overlap with H3K27me3 or not. The group "H3K27me3 marked" represents the genes from the "All" group that were also identified as marked by H3K27me3

in the CUT&Tag experiment. The group “Lost H3K27me3” represents the genes in the “All” group that were not identified as H3K27me3-marked in the CUT&Tag experiment for the corresponding EZH2 mutant-rescued line, but were marked by H3K27me3 in the EZH2 wild type CUT&Tag (i.e. these are genes that lost H3K27me3 upon the mutagenesis of EZH2 and also changed their expression level). For each of these differentially expressed gene groups, “N” represents the number of genes that are up or down regulated. Within each group, the fraction of genes that are upregulated or downregulated are illustrated in cyan and yellow, respectively.

a

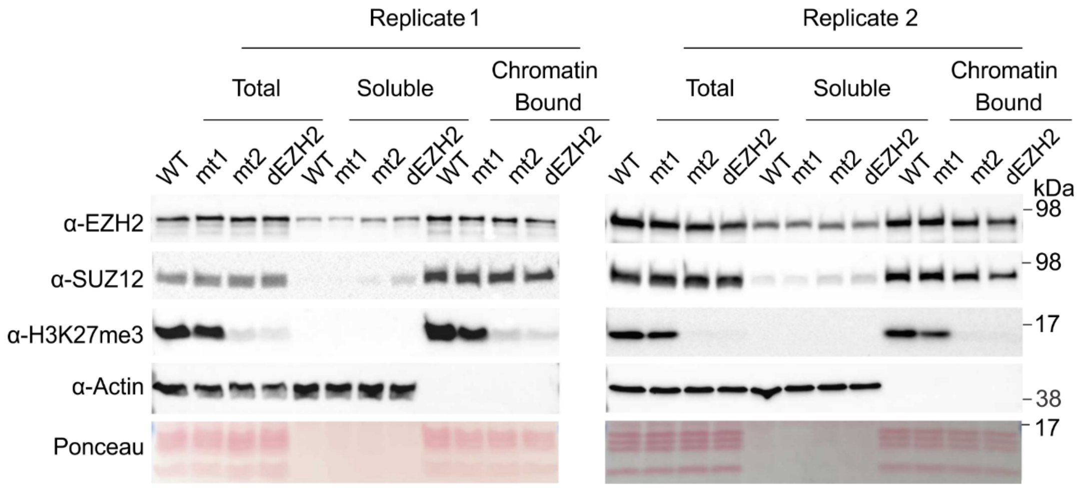

Supplementary Figure 7. A catalytic defective EZH2 mutation leads to lower PRC2 chromatin occupancy compared to the mutations in the nucleic acid-binding surfaces of EZH2.

a. Cellular fractionation of the EZH2 knockout with rescued K562 lines, followed by Western blot analysis using the indicated antibodies. Two biological replicates were carried out and are presented, as indicated.

References

49. Buenrostro, J. D. *et al.* Single-cell chromatin accessibility reveals principles of regulatory variation. *Nature* **523**, 486–490 (2015).

50. Zheng, Y., Ahmad, K. & Henikoff, S. CUT&Tag Data Processing and Analysis Tutorial. *protocols.io* https://www.protocols.io/view/cut-amp-tag-data-processing-and-analysis-tutorial-bjk2kkye (2020).

51. Henikoff, S., Henikoff, J. G., Kaya-Okur, H. S. & Ahmad, K. Efficient chromatin accessibility mapping in situ by nucleosome-tethered tagmentation. *eLife* **9**, e63274 (2020).

52. Jin, H. *et al.* ChIPseqSpikeInFree: a ChIP-seq normalization approach to reveal global changes in histone modifications without spike-in. *Bioinforma. Oxf. Engl.* **36**, 1270–1272 (2020).

53. Ramírez, F., Dündar, F., Diehl, S., Grüning, B. A. & Manke, T. deepTools: a flexible platform for exploring deep-sequencing data. *Nucleic Acids Res.* **42**, W187-191 (2014).

54. Lopez-Delisle, L. *et al.* pyGenomeTracks: reproducible plots for multivariate genomic datasets. *Bioinforma. Oxf. Engl.* **37**, 422–423 (2021).

55. Ramírez, F. *et al.* High-resolution TADs reveal DNA sequences underlying genome organization in flies. *Nat. Commun.* **9**, 189 (2018).

56. Kolde, R. pheatmap: Pretty Heatmaps. (2019).

57. Meers, M. P., Tenenbaum, D. & Henikoff, S. Peak calling by Sparse Enrichment Analysis for CUT&RUN chromatin profiling. *Epigenetics Chromatin* **12**, 42 (2019).

58. Zhu, L. J. *et al.* ChIPpeakAnno: a Bioconductor package to annotate ChIP-seq and ChIP-chip data. *BMC Bioinformatics* **11**, 237 (2010).

59. Patro, R., Duggal, G., Love, M. I., Irizarry, R. A. & Kingsford, C. Salmon provides fast and bias-aware quantification of transcript expression. *Nat. Methods* **14**, 417–419 (2017).

60. Love, M. I., Huber, W. & Anders, S. Moderated estimation of fold change and dispersion for RNA-seq data with DESeq2. *Genome Biol.* **15**, 550 (2014).

61. Soneson, C., Love, M. I. & Robinson, M. D. Differential analyses for RNA-seq:

transcript-level estimates improve gene-level inferences. *F1000Research* **4**, 1521 (2015).

62. Ritchie, M. E. *et al.* limma powers differential expression analyses for RNA-sequencing and microarray studies. *Nucleic Acids Res.* **43**, e47 (2015).

63. Yu, G., Wang, L.-G., Han, Y. & He, Q.-Y. clusterProfiler: an R Package for Comparing Biological Themes Among Gene Clusters. *OMICS J. Integr. Biol.* **16**, 284–287 (2012).

64. Orlando, D. A. *et al.* Quantitative ChIP-Seq Normalization Reveals Global Modulation of the Epigenome. *Cell Rep.* **9**, 1163–1170 (2014).

65. Langmead, B. & Salzberg, S. L. Fast gapped-read alignment with Bowtie 2. *Nat. Methods* **9**, 357–359 (2012).

66. Li, H. *et al.* The Sequence Alignment/Map format and SAMtools. *Bioinforma. Oxf. Engl.* **25**, 2078–2079 (2009).

67. Ramírez, F. *et al.* deepTools2: a next generation web server for deep-sequencing data analysis. *Nucleic Acids Res.* **44**, W160–W165 (2016).
