# Supplementary material for: Inseparable RNA binding and chromatin modification activities of a nucleosome-interacting surface in EZH2
Source: Nat Genet. 2024 May 14;56(6):1193–202. doi: 10.1038/s41588-024-01740-8 (PMC11176075; doi:10.1038/s41588-024-01740-8)
Supplement: Supplementary file 2 — Reporting Summary [file 41588_2024_1740_MOESM2_ESM.pdf]

nature portfolio

Corresponding author(s): Chen Davidovich

Last updated by author(s): Dec 14, 2023

Reporting Summary

Nature Portfolio wishes to improve the reproducibility of the work that we publish. This form provides structure for consistency and transparency in reporting. For further information on Nature Portfolio policies, see our Editorial Policies and the Editorial Policy Checklist.

Statistics

For all statistical analyses, confirm that the following items are present in the figure legend, table legend, main text, or Methods section.

n/a	Confirmed	
☐	☒	The exact sample size (*n*) for each experimental group/condition, given as a discrete number and unit of measurement
☐	☒	A statement on whether measurements were taken from distinct samples or whether the same sample was measured repeatedly
☐	☒	The statistical test(s) used AND whether they are one- or two-sided<br>*Only common tests should be described solely by name; describe more complex techniques in the Methods section.*
☒	☐	A description of all covariates tested
☒	☐	A description of any assumptions or corrections, such as tests of normality and adjustment for multiple comparisons
☐	☒	A full description of the statistical parameters including central tendency (e.g. means) or other basic estimates (e.g. regression coefficient) AND variation (e.g. standard deviation) or associated estimates of uncertainty (e.g. confidence intervals)
☐	☒	For null hypothesis testing, the test statistic (e.g. *F*, *t*, *r*) with confidence intervals, effect sizes, degrees of freedom and *P* value noted<br>*Give P values as exact values whenever suitable.*
☒	☐	For Bayesian analysis, information on the choice of priors and Markov chain Monte Carlo settings
☒	☐	For hierarchical and complex designs, identification of the appropriate level for tests and full reporting of outcomes
☐	☒	Estimates of effect sizes (e.g. Cohen's *d*, Pearson's *r*), indicating how they were calculated

Our web collection on statistics for biologists contains articles on many of the points above.

Software and code

Policy information about availability of computer code

Data collection	Amersham Typhoon Scanner Control software 1.1, PHERAstar 4.00R4, ChemiDoc™ imager, BD Influx™ cell sorter, HiSeq X10, Novaseq 6000
Data analysis	GraphPad Prism 9, SEDNTERP; SEDFIT (16.1c); GraphPad Prism 9; Bowtie2 (2.2.9 and 2.3.5); Picard toolkit (2.26.2); ChIPseqSpikeInFree R package (1.2.4); deepTools (3.1.3 and 3.3.0); pyGenomeTracks (3.5); pheatmap (version 1.0.12); SEACR (1.3); ChIPpeakAnno R package (3.22.4); Salmon (0.14.1); DESeq2 (1.28.1); tximport (1.16.1); limma (3.44.3); clusterProfiler (3.16.1); SAMtools (1.3.1 and 1.9-gcc5); ImageQuant (TL v8.1.0.0.)

For manuscripts utilizing custom algorithms or software that are central to the research but not yet described in published literature, software must be made available to editors and reviewers. We strongly encourage code deposition in a community repository (e.g. GitHub). See the Nature Portfolio guidelines for submitting code & software for further information.

Data

Policy information about availability of data

All manuscripts must include a data availability statement. This statement should provide the following information, where applicable:

- Accession codes, unique identifiers, or web links for publicly available datasets
- A description of any restrictions on data availability
- For clinical datasets or third party data, please ensure that the statement adheres to our policy

CUT&Tag, RNA-Seq and ChIP-Rx data and processed files have been deposited in the NCBI GEO database under accession number GSE239447.

Research involving human participants, their data, or biological material

Policy information about studies with human participants or human data. See also policy information about sex, gender (identity/presentation), and sexual orientation and race, ethnicity and racism.

Reporting on sex and gender	N/A
Reporting on race, ethnicity, or other socially relevant groupings	N/A
Population characteristics	N/A
Recruitment	N/A
Ethics oversight	N/A

Note that full information on the approval of the study protocol must also be provided in the manuscript.

Field-specific reporting

Please select the one below that is the best fit for your research. If you are not sure, read the appropriate sections before making your selection.

☒ Life sciences ☐ Behavioural & social sciences ☐ Ecological, evolutionary & environmental sciences

For a reference copy of the document with all sections, see nature.com/documents/nr-reporting-summary-flat.pdf

Life sciences study design

All studies must disclose on these points even when the disclosure is negative.

Sample size	All experiments were carried out in at least two or three replicates to ensure independent experiments are reproducible, with the exception of ChIP-Rx that was carried out in one replicate. ChIP-Rx were sequenced to a depth of 20 million reads, which is sufficient for analyzing the chromatin binding of PRC2 and the localization of H3K27me3.
Data exclusions	No data were excluded from the analyses.
Replication	See figure legends for information about the number of independent replicates that were carried out for each of the experiments.
Randomization	Randomization was not carried out because different samples included different reagents (e.g. proteins, substrates, antibodies, buffers, etc.) or were subjected to different treatments as indicated, which complicated randomization. Yet, experiments were designed to include controls that allows for objective interpretation of the results.
Blinding	Not performed, as the experiments and data analysis are not subjective.

Reporting for specific materials, systems and methods

We require information from authors about some types of materials, experimental systems and methods used in many studies. Here, indicate whether each material, system or method listed is relevant to your study. If you are not sure if a list item applies to your research, read the appropriate section before selecting a response.

Materials & experimental systems

n/a	Involved in the study	
☐	☒	Antibodies
☐	☒	Eukaryotic cell lines
☒	☐	Palaeontology and archaeology
☒	☐	Animals and other organisms
☒	☐	Clinical data
☒	☐	Dual use research of concern
☒	☐	Plants

Methods

n/a	Involved in the study	
☐	☒	ChIP-seq
☒	☐	Flow cytometry
☒	☐	MRI-based neuroimaging

Antibodies

Antibodies used	anti-Actin (Sigma #A2066, 1:500), anti-EZH2 (Active Motif #39875 for WB with 1:5000 dilution; Cell Signaling #5246 for ChIP-Rx, 6.3

Antibodies used	μg per ChIP), anti-H3 (Abcam #ab1791, 1:50000), anti-H3K27me3 (Cell signaling #9733 for WB with 1:4000 dilution and for CUT&Tag with 1:50 dilution; Cell Signaling #35861SF for ChIP-Rx, 5 μg per ChIP; Active Motif #61017 for WB with 1:2500 dilution), anti-SUZ12 (Santa Cruz Biotechnology #sc-271325 for WB with a dilution of 1:200, Cell signaling #3737S for co-IP with 1.5 μg per IP and ChIP-Rx with 0.5 μg per ChIP), anti-mouse HRP-conjugated (Jackson Immuno-Research #715-035-150, 1:5000), anti-rabbit HRP-conjugated (Santa Cruz Biotechnology #sc2357, 1:5000), anti-H3K27ac (Abcam #4729, 1:50), IgG control (Cell signaling #2729S for CUT&Tag with 1:50 dilution, Cell signaling #3900S for co-IP with 1.5 μg per IP), Guinea pig anti-rabbit antibody (Antibodies online #ABIN101961, 1:100), anti-CBX7 (Abcam #21873, 1:1000), anti-EZH1 (Cell Signaling #42088, 1:1000), anti-H3K27me1 (Merck #07-448, 1:1000), anti-H3K27me2 (Abcam #24684, 1:2000), anti-GAPDH (Proteintech #10494-1-AP, 1:4000).
Validation	All antibodies were commercially available and validated by the manufacture. Anti-Actin (Sigma #A2066) antibody has been validated by Sigma using enhanced antibody validation assay. Anti-EZH2 (Active Motif #39875) antibody has been validated by knocking out EZH2 in our lab, as shown in Fig. 2b (compare the first two lanes of EZH2 blot). The Cell signaling EZH2 antibody (CST #5246) has been validated by Cell Signaling using SimpleChIP Enzymatic Chromatin IP Kit. Anti-H3 antibody (Abcam #ab1791) has been validated by Abcam using western blot. Anti-H3K27me3 (Cell signaling #9733 and #35861SF) were produced from the same clone C36B11, and have been validated by Cell Signaling using SimpleChIP Enzymatic Chromatin IP Kit. Anti-H3K27me3 (Active Motif #61017) has been validated by Active Motif using dot blot. anti-SUZ12 (Santa Cruz Biotechnology #sc-271325) has been validated by Santa Cruz using western blot. anti-SUZ12 (Cell signaling #3737S) has been validated by Cell Signaling using SimpleChIP Enzymatic Chromatin IP Kit. Anti-mouse HRP-conjugated (Jackson Immuno-Research #715-035-150) has been validated by Jackson Immuno-Research using ELISA and/or solid-phase adsorbed. Anti-H3K27ac (Abcam #ab4729) has been validated by Abcam using western blot and ChIP. Guinea pig anti-rabbit antibody (Antibodies online #ABIN101961) has been tested by Antibodies online using ELISA and immunoelectrophoresis assay. Both IgG control (CST #2729 and #3900) have been validated by Cell Signaling using SimpleChIP Enzymatic Chromatin IP Kit. anti-CBX7 (Abcam #ab21873) has been validated by Abcam using western blot. anti-EZH1 (CST #42088) has been validated by Cell Signaling using western blot. anti-H3K27me1 (Merck #07-448) has been tested by Merck using western blot. anti-H3K27me2 (Abcam #24684) has been tested by Abcam using western blot. anti-GAPDH (Proteintech #10494-1-AP) has been validated by Proteintech using western blot, ELISA, FC, IF, IHC, IP.

Eukaryotic cell lines

Policy information about cell lines and Sex and Gender in Research

Cell line source(s)	K562 cells were purchased from ATCC by our lab. HEK293T cells were a gift from Jose Polo lab, Monash University (unknown commercial source). Ezh1-/-;Ezh2f/f;Rosa26CreERT2 mESC line was generated by the lab of Kristian Helin.
Authentication	K562 cells were obtained from ATCC and authenticated by ATCC. HEK293T and Ezh1-/-;Ezh2f/f;Rosa26CreERT2 mESC line were not authenticated.
Mycoplasma contamination	All cell lines are routinely tested for mycoplasma contamination using PCR. The cells used in this work tested negative.
Commonly misidentified lines (See ICLAC register)	No commonly misidentified cell lines were used.

Plants

Seed stocks	*Report on the source of all seed stocks or other plant material used. If applicable, state the seed stock centre and catalogue number. If plant specimens were collected from the field, describe the collection location, date and sampling procedures.*
Novel plant genotypes	*Describe the methods by which all novel plant genotypes were produced. This includes those generated by transgenic approaches, gene editing, chemical/radiation-based mutagenesis and hybridization. For transgenic lines, describe the transformation method, the number of independent lines analyzed and the generation upon which experiments were performed. For gene-edited lines, describe the editor used, the endogenous sequence targeted for editing, the targeting guide RNA sequence (if applicable) and how the editor was applied.*
Authentication	*Describe any authentication procedures for each seed stock used or novel genotype generated. Describe any experiments used to assess the effect of a mutation and, where applicable, how potential secondary effects (e.g. second site T-DNA insertions, mosiacism, off-target gene editing) were examined.*

ChIP-seq

Data deposition

☒ Confirm that both raw and final processed data have been deposited in a public database such as GEO.

☒ Confirm that you have deposited or provided access to graph files (e.g. BED files) for the called peaks.

Data access links *May remain private before publication.*	https://www.ncbi.nlm.nih.gov/geo/query/acc.cgi?acc=GSE239447
Files in database submission	GSM5974936 Ctrl-Ctrl_IgG_cuttag_rp1<br>GSM5974937 Ctrl-Ctrl_H3K27me3_cuttag_rp1<br>GSM5974938 Ctrl-Ctrl_H3K27ac_cuttag_rp1<br>GSM5974939 KO-Ctrl_IgG_cuttag_rp1<br>GSM5974940 KO-Ctrl_H3K27me3_cuttag_rp1<br>GSM5974941 KO-Ctrl_H3K27ac_cuttag_rp1

GSM5974942 KO-WT_IgG_cuttag_rp1
GSM5974943 KO-WT_H3K27me3_cuttag_rp1
GSM5974944 KO-WT_H3K27ac_cuttag_rp1
GSM5974945 KO-mt2_IgG_cuttag_rp1
GSM5974946 KO-mt2_H3K27me3_cuttag_rp1
GSM5974947 KO-mt2_H3K27ac_cuttag_rp1
GSM5974948 KO-mt1_IgG_cuttag_rp1
GSM5974949 KO-mt1_H3K27me3_cuttag_rp1
GSM5974950 KO-mt1_H3K27ac_cuttag_rp1
GSM5974951 KO-dEZH2_IgG_cuttag_rp1
GSM5974952 KO-dEZH2_H3K27me3_cuttag_rp1
GSM5974953 KO-dEZH2_H3K27ac_cuttag_rp1
GSM5974954 Ctrl-Ctrl_IgG_cuttag_rp2
GSM5974955 Ctrl-Ctrl_H3K27me3_cuttag_rp2
GSM5974956 Ctrl-Ctrl_H3K27ac_cuttag_rp2
GSM5974957 KO-Ctrl_IgG_cuttag_rp2
GSM5974958 KO-Ctrl_H3K27me3_cuttag_rp2
GSM5974959 KO-Ctrl_H3K27ac_cuttag_rp2
GSM5974960 KO-WT_IgG_cuttag_rp2
GSM5974961 KO-WT_H3K27me3_cuttag_rp2
GSM5974962 KO-WT_H3K27ac_cuttag_rp2
GSM5974963 KO-mt2_IgG_cuttag_rp2
GSM5974964 KO-mt2_H3K27me3_cuttag_rp2
GSM5974965 KO-mt2_H3K27ac_cuttag_rp2
GSM5974966 KO-mt1_IgG_cuttag_rp2
GSM5974967 KO-mt1_H3K27me3_cuttag_rp2
GSM5974968 KO-mt1_H3K27ac_cuttag_rp2
GSM5974969 KO-dEZH2_IgG_cuttag_rp2
GSM5974970 KO-dEZH2_H3K27me3_cuttag_rp2
GSM5974971 KO-dEZH2_H3K27ac_cuttag_rp2
GSM5974972 Ctrl-Ctrl_IgG_cuttag_rp3
GSM5974973 Ctrl-Ctrl_H3K27me3_cuttag_rp3
GSM5974974 Ctrl-Ctrl_H3K27ac_cuttag_rp3
GSM5974975 KO-Ctrl_IgG_cuttag_rp3
GSM5974976 KO-Ctrl_H3K27me3_cuttag_rp3
GSM5974977 KO-Ctrl_H3K27ac_cuttag_rp3
GSM5974978 KO-WT_IgG_cuttag_rp3
GSM5974979 KO-WT_H3K27me3_cuttag_rp3
GSM5974980 KO-mt2_IgG_cuttag_rp3
GSM5974981 KO-mt2_H3K27me3_cuttag_rp3
GSM5974982 KO-mt2_H3K27ac_cuttag_rp3
GSM5974983 KO-mt1_IgG_cuttag_rp3
GSM5974984 KO-mt1_H3K27me3_cuttag_rp3
GSM5974985 KO-mt1_H3K27ac_cuttag_rp3
GSM5974986 KO-dEZH2_IgG_cuttag_rp3
GSM5974987 KO-dEZH2_H3K27me3_cuttag_rp3
GSM5974988 KO-dEZH2_H3K27ac_cuttag_rp3
GSM5974989 Ctrl-Ctrl_RNAseq_rp1
GSM5974990 KO-Ctrl_RNAseq_rp1
GSM5974991 KO-WT_RNAseq_rp1
GSM5974992 KO-mt2_RNAseq_rp1
GSM5974993 KO-mt1_RNAseq_rp1
GSM5974994 KO-dEZH2_RNAseq_rp1
GSM5974995 Ctrl-Ctrl_RNAseq_rp2
GSM5974996 KO-Ctrl_RNAseq_rp2
GSM5974997 KO-WT_RNAseq_rp2
GSM5974998 KO-mt2_RNAseq_rp2
GSM5974999 KO-mt1_RNAseq_rp2
GSM5975000 KO-dEZH2_RNAseq_rp2
GSM5975001 Ctrl-Ctrl_RNAseq_rp3
GSM5975002 KO-Ctrl_RNAseq_rp3
GSM5975003 KO-WT_RNAseq_rp3
GSM5975004 KO-mt2_RNAseq_rp3
GSM5975005 KO-mt1_RNAseq_rp3
GSM5975006 KO-dEZH2_RNAseq_rp3
GSM5975007 Ctrl-Ctrl_RNAseq_rp4
GSM5975008 KO-Ctrl_RNAseq_rp4
GSM5975009 KO-WT_RNAseq_rp4
GSM5975010 KO-mt2_RNAseq_rp4
GSM5975011 KO-mt1_RNAseq_rp4
GSM5975012 KO-dEZH2_RNAseq_rp4
GSM7665216 1-K562-IN
GSM7665217 2-WT-SUZ12
GSM7665218 3-mt1-SUZ12
GSM7665219 4-mt2-SUZ12
GSM7665220 5-dEZH2-SUZ12

GSM7665221 6-WT-EZH2
GSM7665222 7-mt1-EZH2
GSM7665223 8-mt2-EZH2
GSM7665224 9-dEZH2-EZH2
GSM7665225 10-WT-K27me3
GSM7665226 11-mt1-K27me3
GSM7665227 12-mt2-K27me3
GSM7665228 13-dEZH2-K27me3

Genome browser session (e.g. UCSC)

No longer applicable

Methodology

Replicates

3 independent replicates for CUT&Tag and 1 replicate for ChIP-Rx

Sequencing depth

2.5-23x10^6 reads per sample for CUT&Tag, 2-3x10^7 reads per sample for ChIP-Rx

Antibodies

CUT&Tag: H3K27me3: Cell Signaling Technology #9733; H3K27ac: Abcam #4729; Rabbit IgG control: Cell Signaling Technology #2729; Guinea pig anti-rabbit antibody: Antibodies online #ABIN101961. Primary antibodies: 1:50, secondary antibody: 1:100
ChIP-Rx: Anti-EZH2 (Cell Signalling #5246), 6.3 µg per ChIP. Anti-SUZ12 (Cell Signalling #3737), 0.5 µg per ChIP. Anti-H3K27me3 (Cell Signalling #35861SF), 5 µg per ChIP.

Peak calling parameters

No peaks were called from ChIP-seq data.

Data quality

No peaks were called from ChIP-seq data.

Software

Bowtie2 (2.2.9 and 2.3.5); Picard toolkit (2.26.2); ChIPseqSpikeInFree R package (1.2.4); deepTools (3.1.3 and 3.3.0); pyGenomeTracks (3.5); pheatmap (version 1.0.12); SEACR (1.3); ChIPpeakAnno R package (3.22.4); Salmon (0.14.1); DESeq2 (1.28.1); tximport (1.16.1); limma (3.44.3); clusterProfiler (3.16.1); SAMtools (1.3.1 and 1.9-gcc5)
