# Supplementary material for: Inseparable RNA binding and chromatin modification activities of a nucleosome-interacting surface in EZH2
Source: Nat Genet. 2024 May 14;56(6):1193–202. doi: 10.1038/s41588-024-01740-8 (PMC11176075; doi:10.1038/s41588-024-01740-8)

Fig. 2b

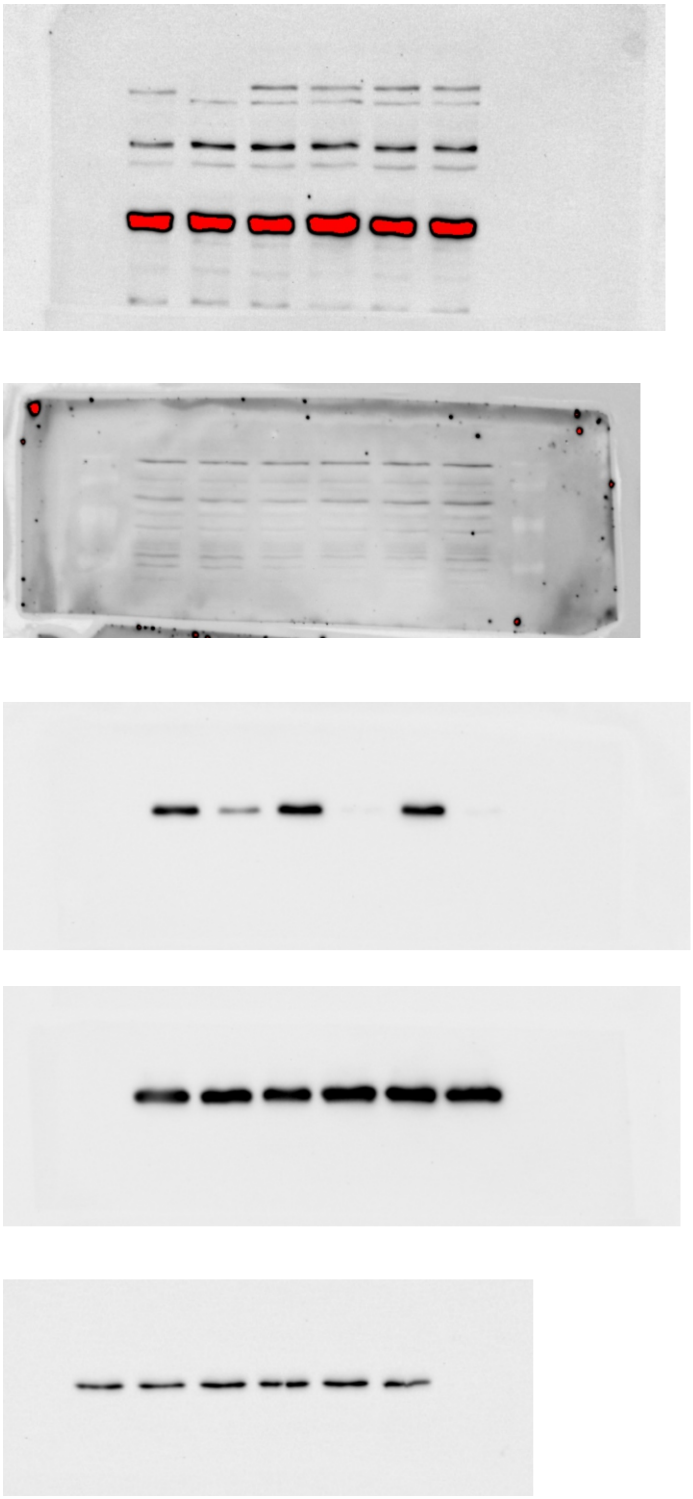

EZH2

SUZ12

H3K27me3

H3

Actin

Fig. S4a

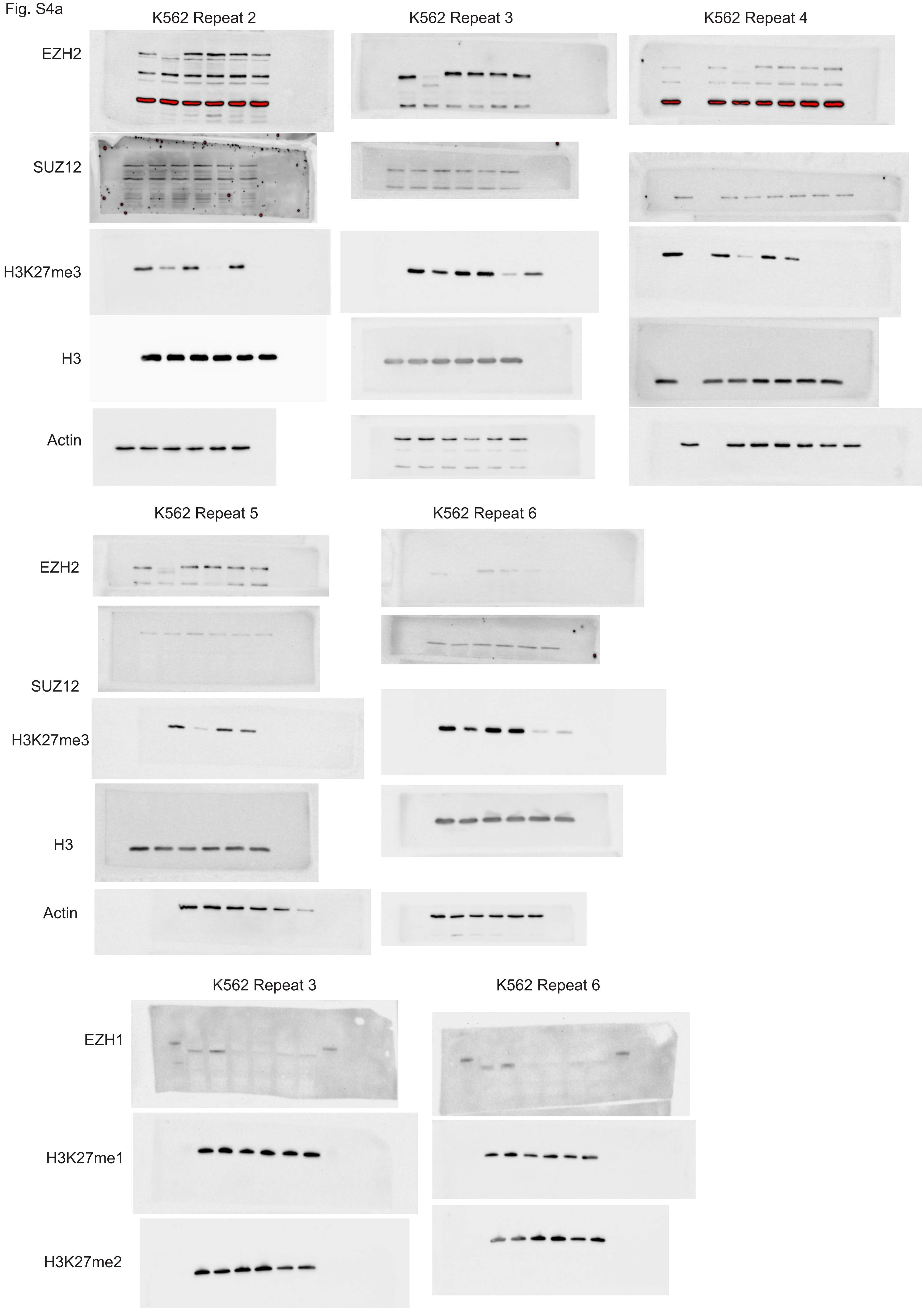
K562 Repeat 2
K562 Repeat 3
K562 Repeat 4
EZH2
SUZ12
H3K27me3
H3
Actin
K562 Repeat 5
K562 Repeat 6
EZH2
SUZ12
H3K27me3
H3
Actin
K562 Repeat 3
K562 Repeat 6
EZH1
H3K27me1
H3K27me2

Fig. S4b

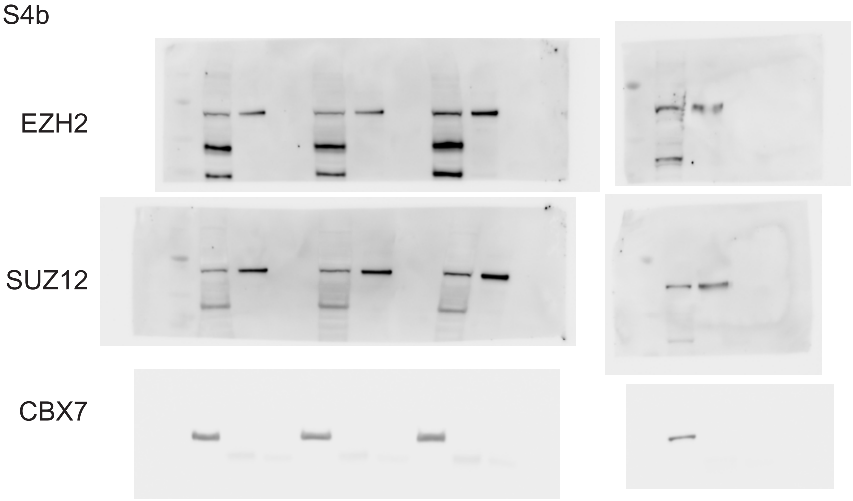

Fig. S4c

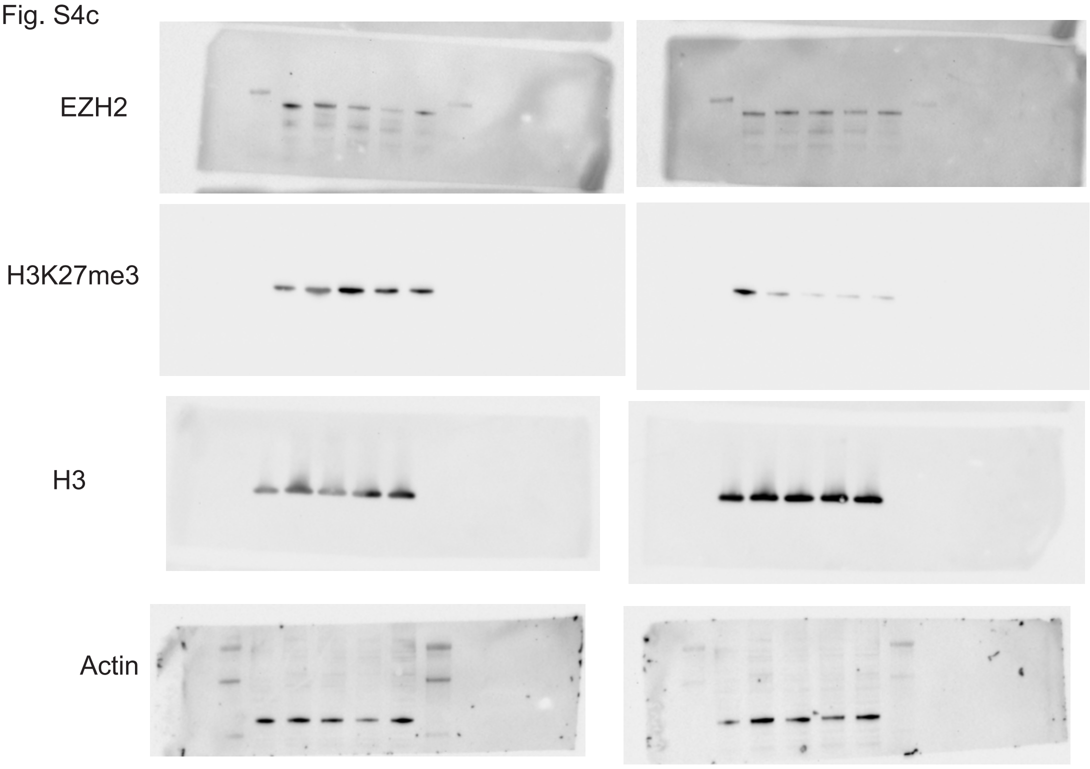

Fig. S4d

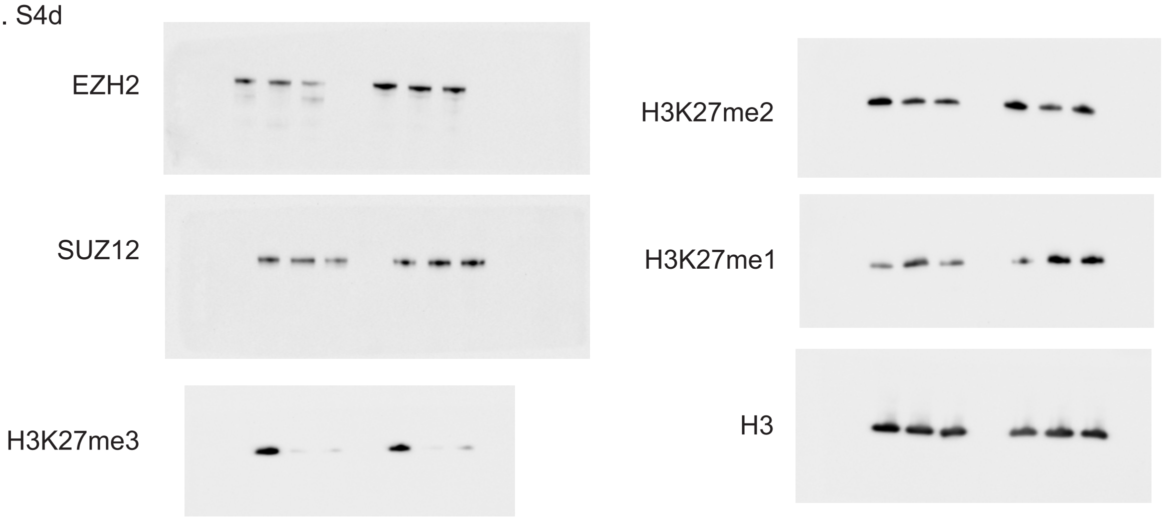

Fig. S7

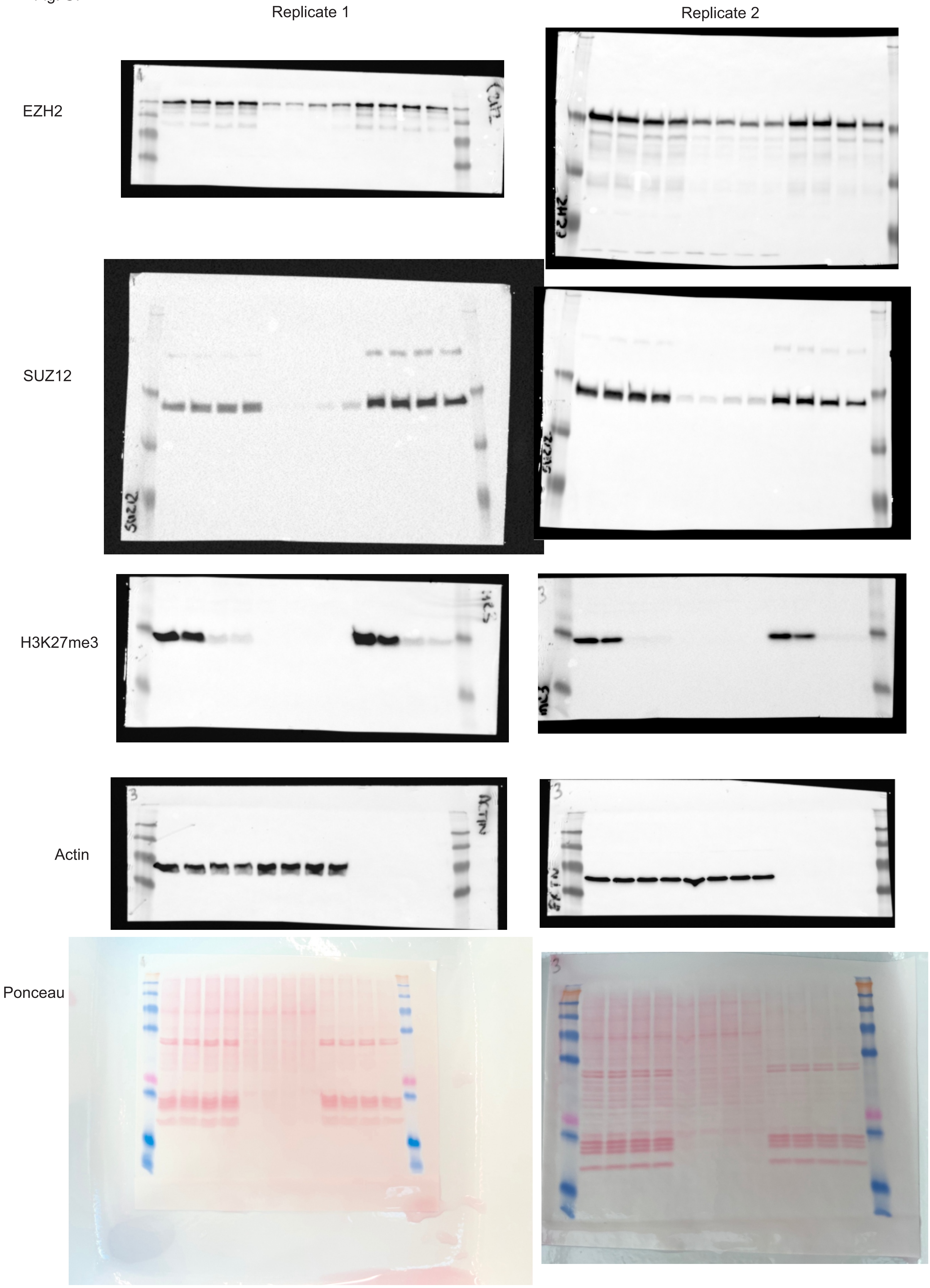

Supplement: Supplementary file 4 — Unprocessed western blots. [file 41588_2024_1740_MOESM4_ESM.pdf]
